# Measuring the Poisson’s Ratio of Fibronectin Using Engineered Nanofibers

**DOI:** 10.1038/s41598-017-13866-3

**Published:** 2017-10-17

**Authors:** John M. Szymanski, Kairui Zhang, Adam W. Feinberg

**Affiliations:** 1 Department of Biomedical Engineering, Carnegie Mellon University, Pittsburgh, PA 15213 USA; 2 Department of Materials Science and Engineering, Carnegie Mellon University, Pittsburgh, PA 15213 USA

## Abstract

The extracellular matrix (ECM) is a fibrillar protein-based network, the physical and chemical properties of which can influence a multitude of cellular processes. Despite having an important role in cell and tissue signaling, a complete chemo-mechanical characterization of ECM proteins such as fibronectin (FN) is lacking. In this study, we engineered monodisperse FN nanofibers using a surface-initiated assembly technique in order to provide new insight into the elastic behavior of this material over large deformations. FN nanofibers were patterned on surfaces in a pre-stressed state and when released from the surface underwent rapid contraction. We found that the FN nanofibers underwent 3.3-fold and 9-fold changes in length and width, respectively, and that the nanofiber volume was conserved. Volume was also conserved following uniaxial extension of the FN nanofibers of ~2-fold relative to the patterned state. This data suggests that the FN networks we engineered formed an incompressible material with a Poisson’s ratio of ~0.5. While the Poisson’s ratio of cells and other biological materials are widely estimated as 0.5, our experimental results demonstrate that for FN networks this is a reasonable approximation.

## Introduction

The extracellular matrix (ECM) is a heterogeneous network of protein-based fibrils that constitutes the physical and chemical properties of the tissue microenvironment and mediates cell processes such as adhesion, differentiation, migration and signalling^1–4^. Fibronectin (FN) is a ubiquitous ECM glycoprotein of particular interest because it is the initial component of the ECM during embryonic development and wound healing, serving as a provisional scaffold for the assembly of other ECM fibrils such as collagen^5^. Structurally, FN is a disulfide-linked, 440 kDa homodimer with multiple binding sites including those for integrins^6^, collagen^5^, heparin^7^, growth factors^8,9^, and self-assembly (polymerization)^10^. Some of these sites are cryptic and require conformational changes of the secondary and/or tertiary structure from a compact, folded state to an extended, unfolded state in order to become exposed and active. In many cases these cryptic sites are exposed under cell-generated forces, forming an insoluble signalling network critical to normal cell communication. Thus, understanding how FN fibrils are mechanically manipulated by cells to expose cryptic domains is critical in developing a mechanistic model of ECM mechanobiology.

Studying the properties of FN fibrils *in vitro* or *in vivo* is challenging because there is a large variability in the length and diameter of cell-derived FN fibers, and these fibers are typically integrated into a more complex ECM containing many other molecules. This limits our ability to study how strain-induced changes in molecular conformation relate to their mechanics and biological function. To address this, researchers have developed several FN fiber fabrication methods that use denaturants^11,12^, surface tension at the air-liquid-solid interface of a FN solution^13,14^, or protein-surface interactions^15,16^ to polymerize FN dimers into insoluble fibers. These studies have demonstrated that through various methods, exposing cryptic self-assembly sites via an induced, conformational change of FN is sufficient to drive fibrillogenesis in a cell-free system.

In this study, we used a surface-initiated assembly (SIA) process in combination with atomic force microscopy (AFM) to engineer FN nanofibers and study their change in morphology over large strains. We hypothesized that FN nanofibers would exhibit elastomeric behavior due to reversible conformational changes of the constituent FN molecules. While previous studies have used tensile testing of FN fibers to demonstrate that they can behave elastically over specific strain regimes^14,17^, key mechanical properties such as Poisson’s ratio remain unknown. Specifically, whether FN can behave as an ideal elastomer with Poisson’s ratio of 0.5 has been difficult to determine because this requires precise measurement of fiber cross-sectional area during tensile strain. To address this, we first used SIA to create an array of FN nanofibers initially patterned as 50 μm long, 20 μm wide rectangles that each easily fit within the AFM scan area. This enabled us to establish that the nanofibers were indeed monodisperse in their dimensions and to then track changes in morphology from the as patterned, pre-release state to the contracted, post-release state. Further, as a second approach we used a modified SIA process to fabricate 1 cm long, 20 μm wide FN nanofibers with regularly spaced fiducial marks, uniaxially strained these nanofibers, and then immobilized these in the strained state and performed AFM to determine morphology. While not identical to cell-generated FN fibers, our results provide key insight into the elastic behavior of insoluble FN networks and how strain-dependent changes in molecular conformation may underlie the mechanobiology of these materials in the native ECM.

## Results and Discussion

### Formation of monodisperse fibronectin nanofibers using surface-initiated assembly

**Figure 1:**
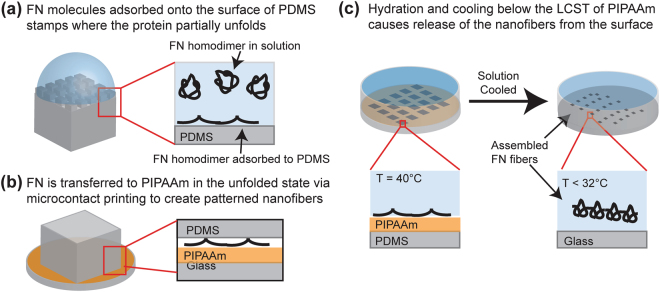
A schematic of the surface-initiated assembly process. (**a**) FN homodimers in their solution conformation were adsorbed onto a hydrophobic PDMS stamp where they obtained a partially unfolded conformation. (**b**) The FN dimers were transferred in the unfolded state to a PIPAAm surface via microcontact printing to create array of patterned nanofibers. (**c**) The PIPAAm was hydrated at an elevated temperature and allowed to cool below its LCST resulting in the release of an assembled FN nanofiber.

Our initial objective was to engineer FN nanofibers that we could morphometrically track during large strains throughout the SIA process in order to quantify volumetric changes and calculate Poisson’s Ratio. However, the technical challenges associated with dynamically tracking nano- or microscale fibers over large strains required us to develop an alternative approach. For example, optical techniques do not have sufficient spatial resolution and AFM cannot accurately image fibers with overhangs, such as those with circular cross-sections immobilized on a surface. To address this, we first engineered monodisperse FN nanofibers with a ribbon-like morphology by modifying a previously published SIA technique^18,19^. Briefly, FN in solution was adsorbed onto a PDMS stamp used for microcontact printing with a surface pattern consisting of 50 μm × 20 μm rectangles (Fig. 1a). The FN was then transferred to a poly(N-isopropylacrylamide) (PIPAAm) substrate through microcontact printing (Fig. 1b). Hydration in 40 °C distilled deionized water (ddH_2_O) and subsequent cooling below the lower critical solution temperature (LCST) of ~32 °C caused the PIPAAm to swell and dissolve and release the assembled FN nanofibers off of the surface into solution (Fig. 1c).

**Figure 2:**
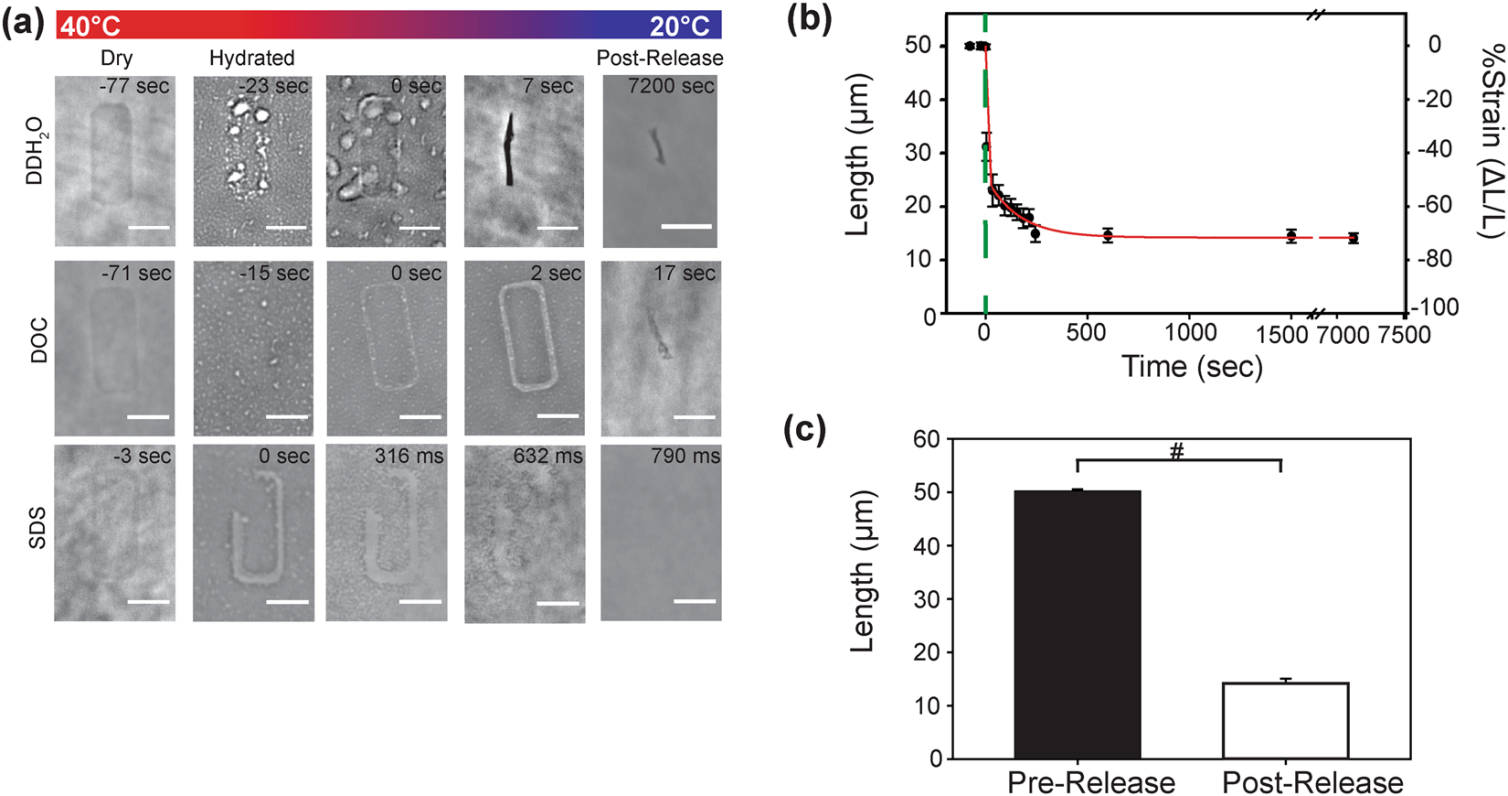
Measuring the dynamic changes in FN nanofiber length during the release process. (**a**) Time lapse sequences of FN nanofibers released in DI water, DOC, and SDS. The times are relative to the initiation of the release from PIPAAm. (**b**) The contour length of FN nanofibers released in DI water. The contour length as a function of time after release (green dashed line) was fit to a double exponential curve (r^2^ = 0.99) with a rapid contraction regime of τ_1_ = 2.88 sec and a slower contraction regime of τ_2_  = 157.41 sec. (**c**) Monodisperse nanofibers 50.12 ± 0.41 μm in length pre-release contracted to a final length of 14.15 ± 0.92 μm, a 3.5-fold decrease in length from the pre-release state Scale bars in (**a**) are 20 μm. Error bars in (**b**) and (**c**) are standard deviation and the symbol ‘#’ indicates a statistically significant difference with P < 0.05.

We used optical microscopy to track the FN nanofibers before, during and after release and quantify dynamic changes in length. In ddH_2_O, using the standard SIA process, the nanofibers in the as patterned, pre-release state rapidly contracted upon release from the PIPAAm surface into a shorter, narrower post-release state (Fig. 2a and supplementary video 1). This contraction after release is consistent with previous reports for SIA generated FN nanofibers^17,18^. Once formed in ddH_2_O, these fibers will remain stable and insoluble in solution for weeks. However, to provide further insight as to how these FN nanofibers might compare to cell-generated FN fibrils, we repeated the release process in either 2% (w/v) deoxycholate (DOC) or 2% (w/v) sodium dodecylsulfate (SDS) solutions (Fig. 2a). Mature FN fibrils are considered to be insoluble in DOC^10,20^, and the FN nanofibers we engineered were found to assemble and remained insoluble when released in DOC. This suggests that SIA of FN forms crosslinks via FN-FN binding sites in a manner similar to FN fibrils in mature cell-derived matrices. In contrast, when released in SDS, the nanofibers immediately dissolved into solution, even before the solution temperature reduced below the LCST of PIPAAm. This suggests that the FN nanofibers were not stabilized by disulphide bonds and instead were cross-linked via non-covalent hydrogen bonding. These results are important because it provides a relative comparison, indicating that the FN nanofibers we engineered are more stable than nascent FN fibrils assembled by cells, but less stable than disulphide-cross-linked FN fibrils that can exist in mature ECM.

### Dynamic release and shape change of fibronectin nanofibers

Tracking the contour length of the FN nanofibers during release in ddH_2_O enabled us to confirm monodispersity throughout the assembly process (Fig. 2b). As soon as the PIPAAm layer began to swell and dissolve, the nanofibers underwent an initial, rapid contraction upon initial release and then proceeded at a slower rate of contraction over a period of several hours. Note that the nanofiber length remained relatively constant as a function of time, indicating that all the nanofibers released and contracted in a similar manner. The contour length was fit to a double exponential curve (r^2^ = 0.99),

$$l(t)={A}_{0}+{A}_{1}{e}^{\frac{-t}{{\tau }_{1}}}+{A}_{2}{e}^{\frac{-t}{{\tau }_{2}}}$$

where the parameters A_o_, A_1_, and A_2_ represent the final contracted length, the decay amplitude, and the fitting parameter, respectively. The FN nanofibers were found to have a fast contraction regime, τ_1_, of 2.88 seconds and a slow contraction regime, τ_2_, of 157.41 seconds. These two rates suggest that FN nanofibers may contract via multiple mechanisms, a rapid entropic-driven contraction and a slower, molecular folding driven contraction. Determining the exact cause is beyond the scope of this manuscript, but will be important to understand in future studies. Comparing the FN nanofiber before and after assembly, we found that the length pre-release was 50.12 ± 0.41 μm, confirming monodispersity (Fig. 2c). Post-release the FN nanofiber contour length was stable after 2 hours at 14.15 ± 0.92 μm, approximately a 3.5-fold decrease in length from the pre-release state.

### Analysis of monodisperse fibronectin nanofiber dimensions and nanostructure using atomic force microscopy

**Figure 3:**
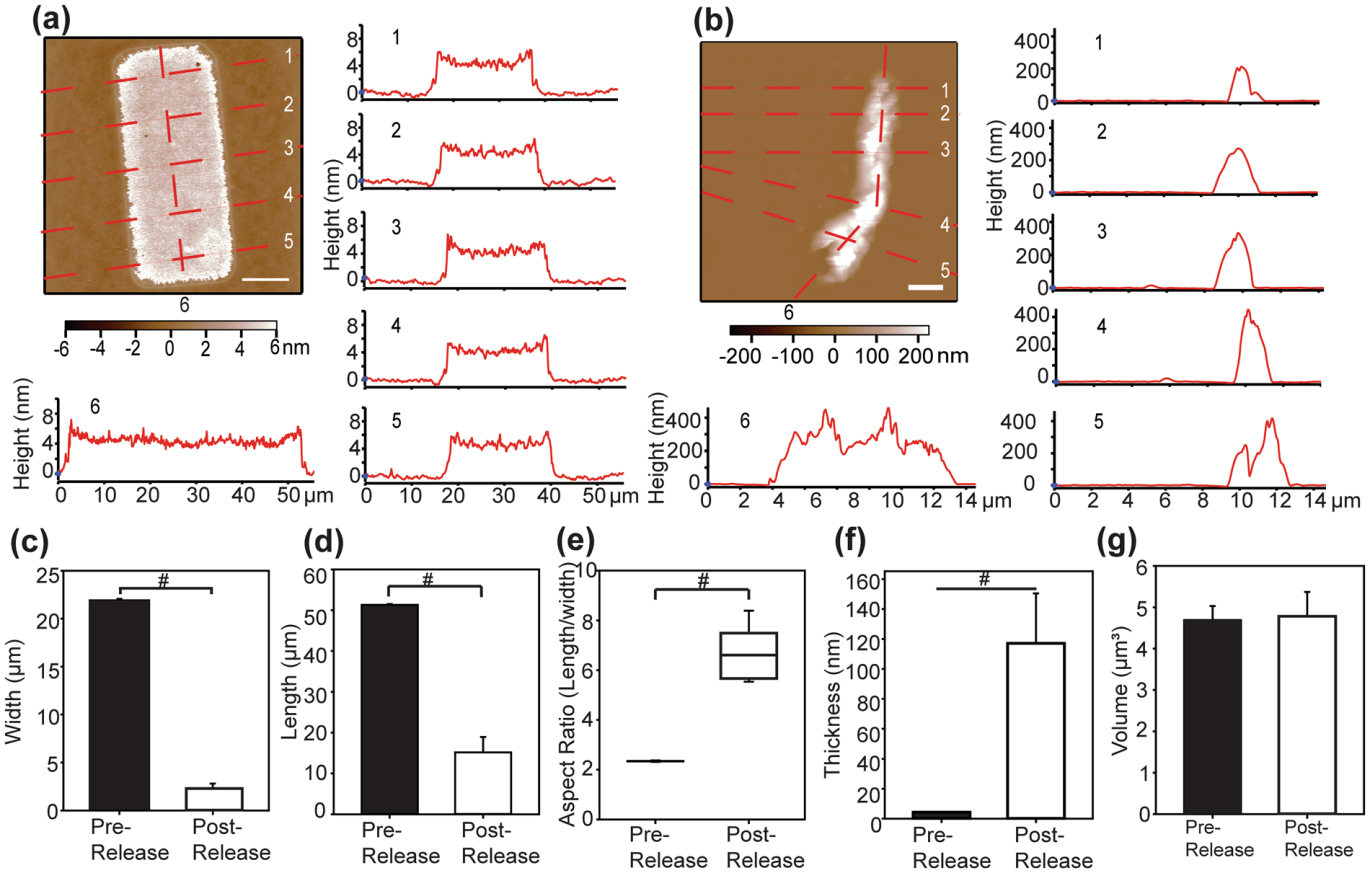
Quantifying morphology of 50 × 20 μm FN nanofibers pre-release and post-release using AFM. (**a**) Pre-release and (**b**) post-release FN nanofibers were measured at five transverse locations to determine the width and longitudinally along the center to determine contour length. (**c**) Nanofiber width was monodisperse and decreased significantly upon release. (**d**) Similarly, nanofiber length was monodisperse and decreased significantly during release. (**e**) The pre-lease aspect ratio of the nanofiber increased significantly post-release due to greater contraction along the width than the length. (**f**) In contrast to the length and width, while nanofiber thickness was monodisperse, it increased significantly during release. (**g**) Nanofiber volume remained constant between pre-release and post-release nanofibers, indicating the FN was incompressible over this strain range. Scale bars are (**a**) 10 μm and (**b**) 2 μm and the symbol ‘#’ indicates a statistically significant difference of P < 0.05.

Next, we used AFM to measure the topography of the FN nanofibers pre-release and post-release. Here the ribbon-like morphology ensured that the AFM could accurately determine the fiber volume from the topographic data and the monodispersity enabled us to image different nanofibers pre- and post-release without having to worry about fiber-to-fiber variability. The FN nanofibers were measured at five transverse locations to determine the mean fiber width and down the middle of the fiber for the contour length for both the pre-release (Fig. 3a) and post-release (Fig. 3b) states. The pre-release and post-release FN nanofibers were monodisperse in width, length and thickness (Fig. 3c,d and f, n = 10), however the dimensional variability, as noted by larger standard deviation, increased for the post-release nanofibers. During release the nanofibers underwent approximately a 9-fold change in width and a 3.3-fold change in length, which corresponds to a change in planar aspect ratio from 2.5 pre-release to 6.6 post-release (Fig. 3g). This anisotropy in contraction appeared to be due to the nanofiber shape, as FN squares created using SIA contracted symmetrically, as previously reported^21^. It should be noted that these AFM measurements were performed in air in order to stabilize the FN nanofibers against a rigid surface to maximize imaging resolution. This dehydration did not alter the post-release length, as the length measured by AFM in air (Fig. 3f) was statistically equivalent to the length measured optically in solution (Fig. 2c) (Mann-Whitney Rank Sum Test, P = 0.42). While this suggests that the FN nanofibers are not a hydrogel^22^, FN based on its amino acid structure does have hydrophilic regions and thus we cannot rule out the possibility other nanofiber dimensions, specifically thickness, may change with hydration.

Using the AFM topographic data we were able to quantify the FN nanofiber volume pre-release and post-release to determine if the large deformation altered material volume. The decrease in nanofiber length and width during release was associated with a large increase in thickness (Fig. 3f). The pre-lease thickness was essentially constant at ~4 nm (Fig. 3a), in agreement with the previously reported thickness of FN nanofibers created by SIA^18^. This thickness is comparable to that reported for FN fibrils formed on polysulfonated substrates^15^ and the diameter for partially-unfolded FN dimers with secondary structure intact^23,24^. In contrast, the post-release thickness was heterogeneous, ranging from approximately 40 nm up to 400 nm (Fig. 3b). This variability was due to the folding of the ribbon-like nanofiber on itself, but this did not interfere with our ability to calculate nanofiber volume. The FN nanofiber volume pre-release was 4.69 ± 0.34 μm^3^ and post-release was 4.78 ± 0.59 μm^3^ (Fig. 3g), statistically equivalent (t-test, P = 0.68). Ideally we would have calculated Poisson’s ratio directly from the changes in length, width and thickness, but the fact that the short nanofibers folded over themselves prevented this. Instead we were able to show that FN nanofibers have a Poisson’s ratio of ~0.5 because they behaved as incompressible, elastic materials that maintained constant volume over large deformations^25–27^. We note that previous studies of FN have shown elastic behavior via tensile testing^14,17^, but it was the use of a monodisperse FN nanofibers and AFM imaging that enabled us to accurately measure pre- and post-release volumes and determine that they are incompressible.

**Figure 4:**
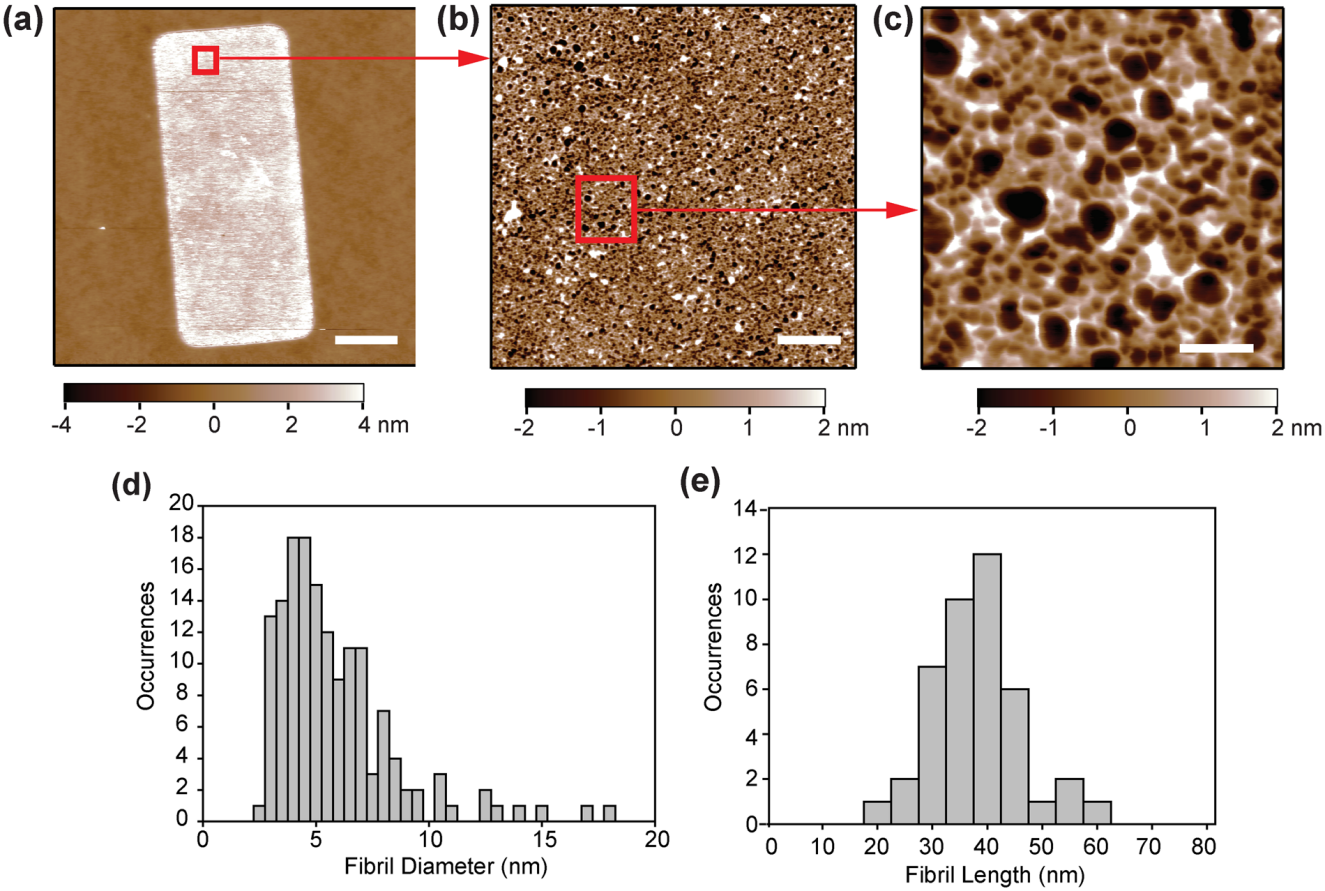
FN nanofibers pre-release exhibit a fibrillar nanostructure. (**a**) An AFM scan of an entire FN nanofiber in a pre-release state on PIPAAm. (**b**,**c**) Increasing the resolution by sequentially scanning sub-regions of (**a**) revealed a fibrillar nanostructure consisting of nodes interconnected by linear fibrils. (**d**) The linear fibrils between intersection points had an average width of 5.72 ± 2.64 nm although fibrils with widths as small as ~3 nm and as large as ~17 nm were also observed. (**e**) The length of the linear fibrils was measured to be 35.51 ± 7.49 nm. Scale bars are (**a**) 10 μm, (**b**) 1 μm, and (b) 100 nm.

Next, we examined the nanostructure of the FN nanofibers to better understand how changes in molecular conformation may be contributing to the contraction during release. In the pre-release state the FN nanofibers were comprised of an isotropic, network of interconnected fibrils (Fig. 4a–c), comparable to the nanostructure previously reported in pre-release FN and laminin nanofibers formed using SIA^18,28^. The smallest resolvable features observed were linear fibrils that had an average width of 5.72 ± 2.64 nm, although fibrils as small as ~3 nm were also observed (Fig. 4d). Since the width of a single FN dimer in an unfolded state without the loss of secondary structure is ~2–3^23,24^, we reasoned that these fibrils were typically 1–3 unfolded FN dimers in parallel. Next, we measured the length of the linear fibril segments between intersection points and observed an average length of 35.51 ± 7.49 nm (Fig. 4e). This length is much less than FN dimers that have lost their tertiary structure but retained secondary structure of the type III repeats, estimated to have a contour length of ~120–160 nm^23,24^. This suggests that the intersection points contain overlapping FN dimers in an unfolded state. It is likely that this enables the formation of the interconnected FN molecular networks we observe by AFM and the reason that we maintain an insoluble FN nanofiber upon dissolution of the PIPAAm layer.

**Figure 5:**
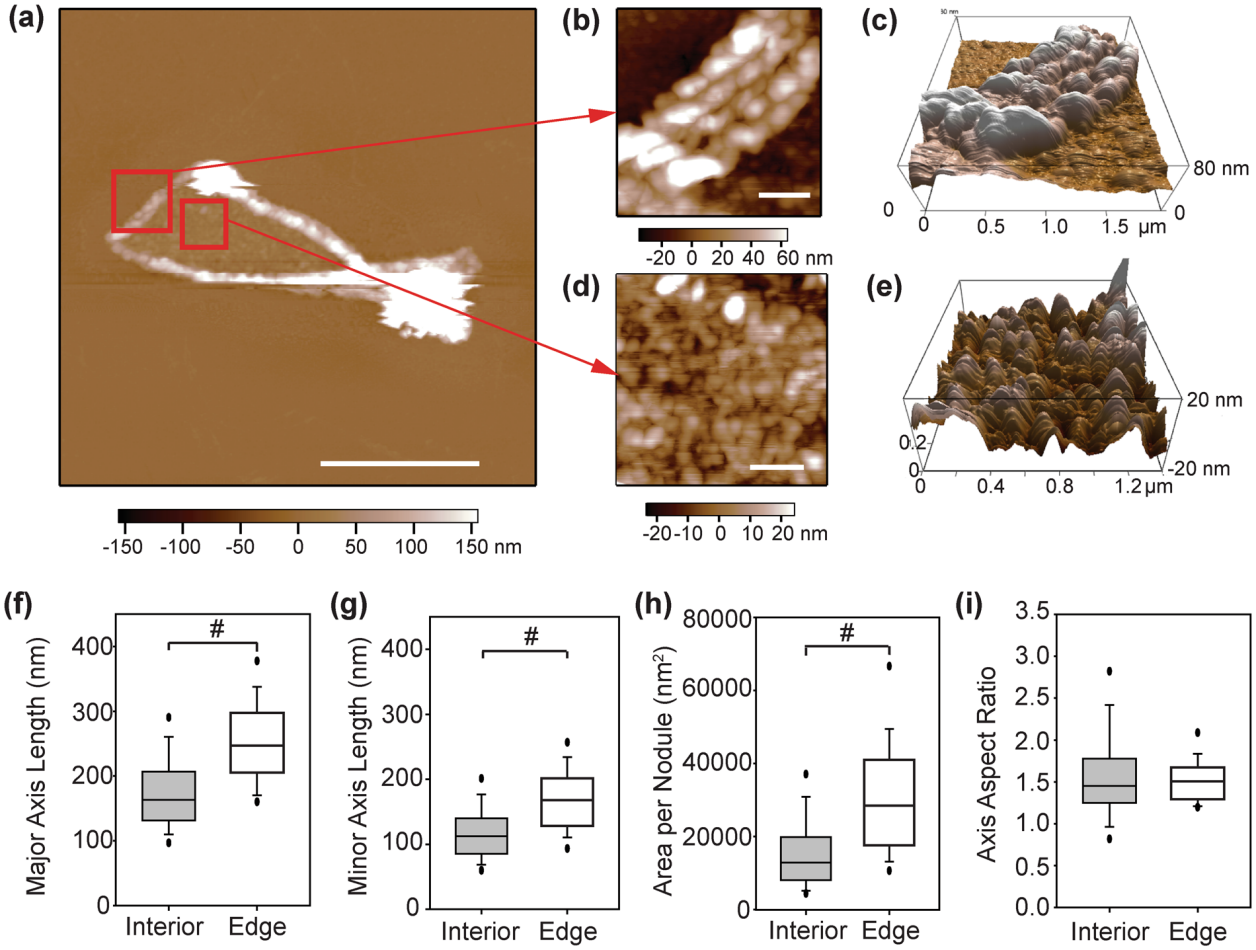
FN nanofibers post-release have a nodular nanostructure. (**a**) An AFM scan of an entire FN nanofiber post-release. (**b**) The outer edge region contained elliptically-shaped nodules ~80 nm in height that extended 1–1.5 μm inward from the perimeter of the nanofiber. (**c**) The interior region contained a similar nodular structure although the nodules were smaller and ~40 nm in height. The nodules in the edge region had a significantly larger (**d**) major axis and (**e**) minor axis length than the nodules found in the interior region. (**f**) Comparable to the axis lengths, the cross-sectional area of the nodules in the edge region was statistically greater than the interior nodules. (**g**) Despite the differently sized nodules, the aspect ratio was consistent, suggesting a similar packing of elliptical dimers within the nodules. Scale bars are (**a**) 5 μm and (**b** and **d**) 500 nm. The symbol ‘#’ indicates a statistically significant difference with P < 0.05.

In comparison, the post-release FN nanofibers had distinctly different nanostructure. We imaged post-release FN nanofibers in areas where they were not folded over on themselves (Fig. 5a), and observed that they were comprised of elliptically shaped nodules on the order of several hundred nanometers in width (Fig. 5b–e). Interestingly, there were structural differences between nodules found at the outer edge of the nanofiber (Fig. 5b,c) versus nodules found in the inner region (Fig. 5d,e). Careful observation of the nanofiber release process showed that the outer edge of the nanofiber began to rapidly contract first, before the interior contracted more slowly (Fig. 2a). This temporal difference may account for the difference in nodule size. All the nodules had an overall elliptical morphology with a major axis length of 173.69 + 57.84 nm (n = 239) and 254 + 65.32 nm (n = 102) for the interior and edge nodules, respectively (Fig. 5f). Similarly, the minor axis length was 117.15 + 42.03 nm and 169.94 + 49.40 nm for the interior and edge nodules, respectively (Fig. 5g). Based on major and minor axis length, the cross-sectional area of the nodules on the edge were significantly greater than the interior (Fig. 5h), suggesting more FN dimers were folded within each nodule. FN dimers in the compact solution conformation are ellipsoidal with known dimensions of 51 × 32 nm^24,29^, similarly suggesting that the nodules observed here were comprised of multiple folded FN dimers. Despite differences in nodule size between the two regions, the aspect ratio remained effectively constant at 1.59 ± 0.63 and 1.53 ± 0.30 for the interior and edge regions, respectively (Fig. 5i). Interestingly, this aspect ratio is consistent with the aspect ratio reported for FN dimers in their compact solution conformation^29^, suggesting that as multiple dimers fold into nodules within the nanofiber they may retain some of this solution-like conformation.

### Analysis of uniaxially strained fibronectin nanofiber dimensions and nanostructure using atomic force microscopy

**Figure 6:**
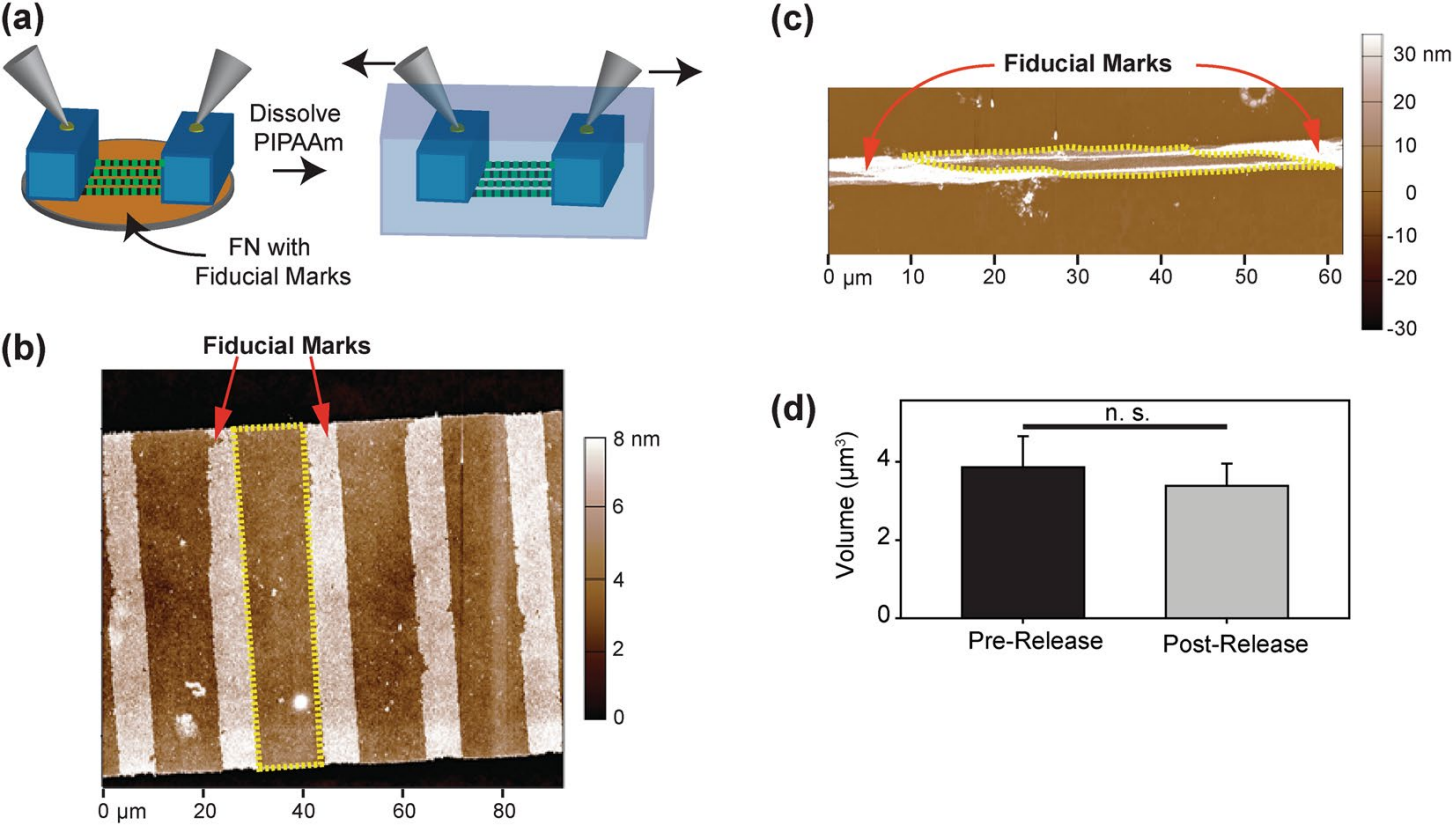
Quantifying the volumetric changes in strained FN nanofibers. (**a**) FN nanofibers were strained by releasing the FN nanofibers onto PDMS supports and uniaxially stretching them using micromanipulators. (**b**) An AFM scan of a ~67 μm wide FN nanofiber in a pre-release state on PIPAAm. The fiducial marks added an extra FN layer to the FN nanofibers and thus appear white in the AFM images. The volume was measured in the regions between fiducial marks (red outline). (**c**) After being strained to ~98%, the FN nanofiber was immobilized in the strained state and scanned using AFM. The volume was measured in the regions between fiducial marks (yellow dotted outline). (**d**) Quantification of FN nanofiber volume indicates there was no statistically significant difference.

Finally, to validate whether FN nanofibers maintained incompressible behavior over tensile strains, we developed an approach to perform uniaxial tensile testing on the FN nanofibers (Fig. 6). To do this we engineered FN nanofibers that were ~67 μm wide and 1 cm long. The FN nanofibers were first tagged with FN-based fiducial marks through a modification of a previously published Patterning-on-Topography method (Supplementary Figure 1)^30,31^. These fiducial marks consisted of a second layer of FN patterned at defined intervals longitudinally along the FN nanofibers, and could be clearly observed in phase-contrast, fluorescent and atomic force microscopies. The nanofibers were released onto PDMS supports where they could be uniaxially stretched using a pair of micromanipulators (Fig. 6a). To measure strain, we calculated the center-to-center distance of consecutive fiducial marks in both the pre-release and tensile strained states. FN nanofibers pre-release on PIPAAm had a center-to-center fiducial mark distance of 20.21 ± 0.68 μm (Fig. 6b, n = 3). When released, strained, and immobilized for AFM imaging, we measured an average fiducial mark center-to-center distance of 40.06 ± 8.68 μm (Fig. 6c, n = 5) representing an average strain of 98.2%. When we measured the volume of the FN segments between the fiducial marks, we found that the volume decreased slightly from 3.86 ± 0.79 μm^3^ to 3.38 ± 0.57 μm^3^ (Fig. 6d), however, there was no statistical difference between the pre-release and strained FN nanofiber volumes (t-test, P = 0.35). This provided experimental evidence that, similar to the smaller monodisperse FN nanofibers, the FN nanofibers subjected to ~100% uniaxial tensile strain from the pre-release state also behaved as an incompressible material.

### Analysis of fibronectin nanofiber deformation over the entire strain regime

Taken together, the results from the contraction of the short monodisperse nanofibers (Fig. 3) and tensile strain of the long FN nanofibers (Fig. 6) demonstrated that the FN nanofibers conserved volume over very large deformation. Since both measurements were done relative to the pre-release state, we combined the results from Figs 3 and 6 to estimate the entire deformation range over which FN has this elastic behavior behavior. Specifically, the short monodispersed FN nanofibers decreased in length from the pre-release state by 3.3-fold and the uniaxial strained FN nanofibers increased in length from the pre-release state by 2-fold. This corresponds to a > 6.5-fold stretch ratio from the full contracted state to the uniaxially tensile strained state, providing strong experimental evidence that the Poisson’s ratio is 0.5 across this regime. Altogether, we reasoned that FN homodimers in the pre-release nanofibers had lost their tertiary, globular structure and thus were fibrillar, but upon release the FN homodimers attempted to regain their tertiary structure and refold. However, the nodules observed in the post-release nanofibers were much larger than single FN homodimers in solution (~50 nm)^24,29,32^, suggesting that these nodules included many molecules folded up together into a more complex structure. A more in-depth analysis is beyond the scope of this current study, but clearly a better understanding of how FN molecular conformation changes as a function of strain and the impact this has on FN mechanobiology is critically important.

## Conclusions

We have demonstrated the ability to engineer monodisperse FN nanofibers that can be used to better understand the mechanical behavior of insoluble FN networks undergoing large deformation. Despite multi-fold changes in the length, width, and, thickness, we show using AFM analysis that the volume was conserved. This enables us to estimate that the FN nanofibers have a Poisson’s ratio of ~0.5, which had not been previously validated experimentally. Further, we showed that the changes in nanofiber morphology from the pre-release to post-release states were associated with a change in the nanostructure of the FN molecular network from fibrillar to nodular. It is important to note that FN is only a single component in a far more complex ECM, and thus we plan to investigate the mechanical behavior of other ECM proteins such as laminin and collagen type IV, as well as integrated networks of these proteins, as we work to engineer nanofibers with increasing complexity and physiologic relevance.

## Methods

### Fibronectin nanofiber fabrication

FN nanofibers were prepared via an SIA technique based on previously described^18^. Briefly, PDMS stamps patterned with 50 × 20 μm raised rectangles were fabricated by first spincoating glass wafers with SPR 220.3 positive photoresist (Microchem). The photoresist was then exposed to UV light through a photomask and developed using MF-319 developer (Microchem). A negative of the patterned photoresist wafer was formed by casting PDMS prepolymer (Sylgard 184, Dow Corning) over it and curing it at 65 °C for 4 hours. The cured PDMS was then peeled off the glass wafer and 1 cm^2^ stamps were cut out.

Prior to use, the PDMS stamps were sonicated in a 50% ethanol solution for 30 minutes and then dried under a stream of nitrogen. The stamps were then incubated with FN (BD Biosciences) at a concentration of 50 μg/ml in ddH_2_O water for 30 minutes, washed to remove excess FN, and then dried under stream of nitrogen. FN coated PDMS stamps were then brought into conformal contact with PIPAAm coated coverslips for 10 minutes to create arrays of 50 × 20 μm FN rectangles on the PIPAAm surface. The PIPAAm coated coverslips were prepared by spincoating a 10% PIPAAm (Polysciences Inc.) in 1-butanol (w/v) solution. Upon removal of the stamps, the quality of the FN rectangles on the PIPAAm was inspected using phase contrast microscopy. The FN patterns were released from the PIPAAm surface by adding warm, ~40 °C, ddH_2_O and allowing the temperature to gradually drop below the LCST of PIPAAm, resulting in the dissolution of the PIPAAm layer and the non-destructive release of the nanofibers. To test detergent solubility, the nanofibers were released in either a solution containing 2% (w/v) sodium dodecyl sulphate dissolved in ddH_2_O or a solution containing 2% (w/v) sodium deoxycholate dissolved in ddH_2_O.

### Dynamic change in nanofiber morphology

The FN nanofiber release was monitored optically using phase contrast microscopy and time-lapse images were captured using a Photometrics CoolSnap ES digital camera (1392 × 1040 pixels) at a frame rate of 1 fps. The contour length of 15 nanofibers was measured pre-release (−77 and −23 sec), at the initiation of release (0 sec), and post-release from the PIPAAm surface (4, 30, 60, 90, 120, 150, 180, 210, 240, 600, 1500, and 7200 sec) using ImageJ (National Institutes of Health)^33^.

### Atomic force microscopy of nanofiber morphology

AFM (MFP3D-Bio, Asylum Research) was used to analyze the nanostructure and quantify the morphology of FN nanofibers both pre-release and post-release. The FN nanofibers were scanned in air using AC mode with AC160TS cantilevers (Olympus Corporation). Nanofibers pre-release were scanned on the PIPAAm surface whereas post-release FN nanofibers were first allowed to adsorb back onto the glass coverslip, washed three times with ddH_2_O to remove the dissolved PIPAAm, and then dried in a 65 °C oven. FN nanofibers pre-release were scanned with a scan size of 512 × 512 lines over a scan area of ~52 μm × 52 μm and FN nanofibers post-release were scanned with a scan size of 512 × 512 lines over a scan area of ~15 μm × 15 μm. High-resolution AFM images were obtained using a scan size of 1024 × 1024 lines over a scan area of 3 μm × 3 μm for the nanofibers pre-release (zoomed in to a 500 × 500 nm area after acquisition) and 2 × 2 μm for nanofibers post-release.

### Uniaxially Stretching FN Nanofibers

To strain FN nanofibers we used our previously published technique^31^. We first microcontact printed 1 cm long and 67 μm wide lines of FN that have been tagged with fiducial marks (Supplementary Figure 1). Next, we prepared PDMS pads by casting PDMS prepolymer over a glass slide and curing it in an oven set to 65 °C for 4 hours. Once cured we cut out 2 PDMS rectangles that were ~1 cm long and ~5mm wide and placed them at opposite ends on top of the FN nanofibers that were microcontact printed on PIPAAm. We then attached the PDMS pads to a pair of micromanipulators by applying a small drop of high strength epoxy (Devcon) to the top of the pads, bringing the manipulator tips in contact with the epoxy, and allowing the epoxy to harden for at least 30 minutes. Once the epoxy hardened, we triggered the thermal dissolution of PIPAAm by hydration and subsequent cooling below the LCST of PIPAAm, which led to the assembly and release of the nanofibers off of the PIPAAm and onto the PDMS pads. This resulted in FN fibers that were tethered at each end to a PDMS pad and freely suspended in between. The FN nanofibers were strained to ~98% by moving the micromanipulators in opposite directions and subsequently immobilized back onto the coverslip. To calculate strain, we measured the center-to-center fiducial mark distance of FN nanofibers in a pre-release state as the initial length and the center-to-center fiducial mark distance of strained FN nanofibers as the final length. These strain experiments were performed on top of an inverted epifluorescent microscope (Eclipse Ti, Nikon Instruments) with mounted micromanipulators (Transferman NK2, Eppendorf).

### Data analysis and statistical methods

To accurately quantify FN nanofiber morphology, the AFM height signal of each nanofiber was first processed to ensure the background surface was flat and centred at 0 nm. To accomplish this, the FN nanofibers in the image were masked and the non-masked area was subject to a 1^st^ order planefit and a 2^nd^ order flattening. The width at five transverse locations and contour length for ten nanofibers were then measured using the IGOR Pro software environment. FN nanofiber volume and thickness were also measured using the IGOR Pro software environment. The FN nanofiber volume was calculated by first applying a mask to the FN nanofibers. The volume of each pixel within the confines of the mask was calculated by multiplying the AFM height value of a given pixel by its area. The total FN nanofiber volume was measured as the summation of the volume of each pixel within the masked area. For FN nanofibers that were uniaxially stretched, only the regions of the nanofibers between fiducial marks was masked and the volume was measured as previously described. Since thickness was heterogeneous along the length and width of the nanofibers (Fig. 3a and b), the average fiber thickness was quantified by dividing the volume of the nanofibers by its area. The Mann-Whitney rank sum test with P < 0.05 was used for statistical analysis unless otherwise noted (Sigma Plot, Systat Software Inc.).

## Electronic supplementary material

Supplementary Information

Supplementary Video 1
