# Supplementary material for: Measuring the Poisson’s Ratio of Fibronectin Using Engineered Nanofibers
Source: Sci Rep. 2017 Oct 17;7:13413. doi: 10.1038/s41598-017-13866-3 (PMC5645409; doi:10.1038/s41598-017-13866-3)
Supplement: Supplementary file 1 — Supplementary Information [file 41598_2017_13866_MOESM1_ESM.pdf]

Supplementary Information

Measuring the Poisson's Ratio of Fibronectin Using Engineered Nanofibers

John M. Szymanski[1], *Kairui Zhang*[1] and Adam W. Feinberg[1,2]*

[1]*Department of Biomedical Engineering, Carnegie Mellon University, Pittsburgh, PA 15213*

[2]*Department of Materials Science and Engineering, Carnegie Mellon University, Pittsburgh, PA 15213*

* Corresponding author: feinberg@andrew.cmu.edu

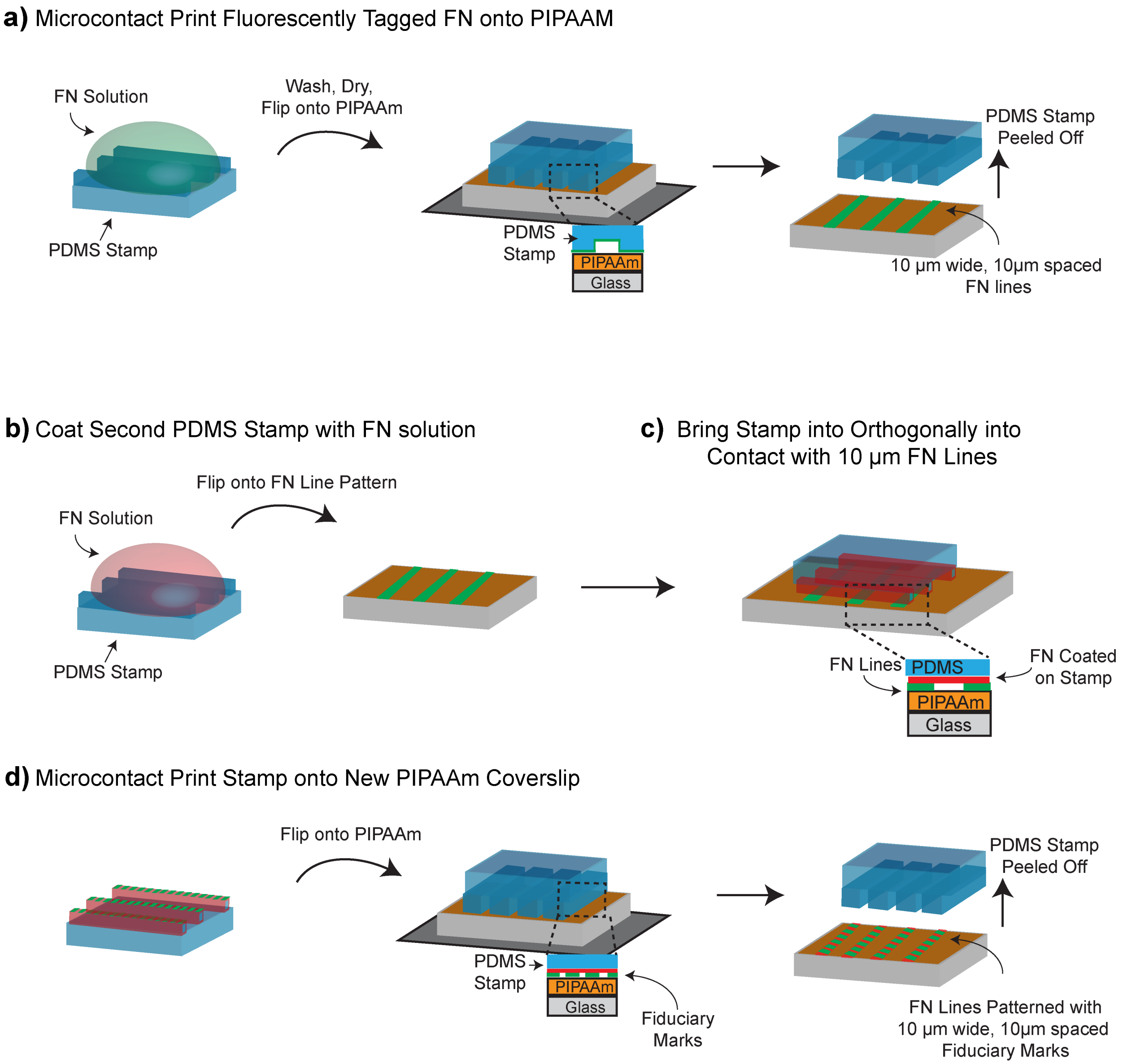

Supplementary Figure 1. Fabrication of FN nanofibers with fiduciary marks. (a) First, 10 µm wide FN lines (green) were microcontact printed onto a PIPAAm coated coverslip. (b) Second, a PDMS stamp coated with FN and dried (red) was (c) brought into conformal contact orthogonal to the 10 µm wide FN lines on the PIPAAm. The 10 µm wide FN lines were released and conformally transferred onto the FN coated stamp by triggering the dissolution of the PIPAAm, known as patterning on topography (PoT). (d) Finally, the FN coated (red) PDMS stamp with 10 µm wide orthogonal FN lines (green) was microcontact printed onto a new PIPAAm coated coverslip to create FN nanofibers with 10 µm wide, 10 µm spaced fiduciary marks.

Supplementary Video 1. Surface-initiated assembly of FN nanofibers. FN was microcontact printed onto PIPAAm as rectangles with planar dimensions of 50 µm x 20 µm. The PIPAAm was then hydrated in ddH_2O at 40 °C. Upon reduction of temperature below the lower critical solution temperature (LCST) of PIPAAm (~32° C), the nanofibers released from the surface and rapidly contracted.
